# Theta oscillations are associated with movement during choreographed and improvised dance - a case series with Memphis Jookin’: The Show

**DOI:** 10.21203/rs.3.rs-8398609/v1

**Published:** 2026-01-05

**Authors:** Noor Tasnim, Alana Hutchinson, Daphne Gyamfi, Krishna Makani, Grace Nobriga, Julia Basso

**Affiliations:** Virginia Tech; Virginia Tech; Virginia Tech; Virginia Tech; Virginia Tech; Virginia Tech

**Keywords:** Flow, EEG, Connectivity, Dance, Theta, Interoception

## Abstract

Flow is a psychological state of deep immersion and engagement associated with enhanced performance and well-being, yet its neural correlates remain poorly understood. Here, we investigated whether flow is experienced during Memphis Jookin’, a street dance style originating in Memphis, Tennessee, and examined its behavioral and neurophysiological signatures. Professional Memphis Jookers (N = 6) completed validated self-report measures assessing flow, interoceptive awareness, and embodied responses to movement. Two participants wore 32-electrode electroencephalography (EEG) systems while engaging in choreographed and improvised dance, observing other dancers, and resting state. Independent components were localized using dipole modeling for one participant, with spectral parameterization and functional connectivity assessed. Dancers reported high levels of trait and state flow and demonstrated elevated interoceptive awareness compared to individuals trained in other mind–body practices. Theta-band activity was prominent during dance across regions including the posterior cingulate gyri, inferior temporal gyrus, middle occipital gyrus, paracentral lobule, supplementary motor area, and Rolandic operculum. Resting-state functional connectivity increased after dance across theta, alpha, beta, and gamma frequency bands. Together, these findings suggest that street dance elicits robust flow states accompanied by distinct patterns of large-scale brain activity and connectivity, highlighting dance as an embodied practice with translational relevance for health and well-being.

## Introduction

Scientists, artists, philosophers, and anyone working to accomplish a goal often seek to find moments of “being in the zone” - to achieve a state of flow. Flow is the psychological state of being completely immersed in the moment, fully engaged, and with the ability to pay attention for prolonged periods of time (Csikszentmihalyi, 2014). In this state, time may become distorted and the individual may have a sense of being completely in the moment such that intention, action, and awareness become one. In cognitive neuroscience, this state has been termed effortless attention (Dietrich & Stoll, 2010). Flow has traditionally been examined in individuals at the peak of their professions including athletes (S. A. Jackson, 1995) and performers (Jaque et al., 2020; Wrigley & Emmerson, 2013); however, flow has also been examined during everyday experiences, including work (Fullagar & Kelloway, 2009) and video game playing (Soutter & Hitchens, 2016).

Though the neurocognitive processes of flow remain largely unknown, recent studies have begun to explore the brain’s role in flow (Gold & Ciorciari, 2020). A handful of these studies have been guided by two hypotheses that explain the role of the brain’s various networks in regulating flow. The Transient Hypofrontality Hypothesis argues that activity from the prefrontal cortex decreases during flow and reduces top-down attentional control (Dietrich, 2003). Rather, as movement becomes more automatic, neural engagement is thought to shift towards greater engagement of the basal ganglia (Poldrack et al., 2005). In contrast, Weber’s synchronization theory argues that flow requires attention to stay focused on a task, and thus, flow is associated with a harmonious synchronization across alerting, orienting, and executive attention networks (Weber et al., 2009). Moreover, Weber argues that these attention networks cooperate with reward pathways in the brain to lead to flow (Weber et al., 2009). Dopamine is a well-known neurotransmitter associated with these reward pathways (Schultz, 2006) and studies have found that individuals with more dopamine D2 receptors in their striatum are more likely to experience flow (Nieoullon, 2002).

In practice, functional magnetic resonance imaging studies have revealed a pattern of activation and deactivation in several distinct brain regions. Namely, brain regions that code for outcome probability (putamen) and cognitive control (inferior frontal gyrus) show increased activation, whereas brain regions involved in self-referential processing (medial prefrontal cortex) and emotional arousal (amygdala) exhibit decreased activation (Ulrich et al., 2014). This deactivation of default mode network (DMN) regions is noteworthy, as flow states are often characterized by a diminished awareness of oneself as a social actor. As a result, self-consciousness decreases, which would be an expected psychological corollary of a decrease in DMN activation.

However, flow is often a psychological state that emerges during whole-body movement through time and space, which is impossible to do in a brain scanning scenario where the individual needs to be completely sedentary without movement of the head. As a result, few studies have employed recent advancements in electroencephalography (EEG) to understand brain states associated with flow. For example, EEG has been used to understand flow state during gaming (Rácz et al., 2025), music playing (Tan et al., 2024), and computerized executive function tasks (Katahira et al., 2018). Some of these studies have suggested that brain activity in the frontal regions of the brain decreases during flow, in support of the Transient Hypofrontality Hypothesis; however, experimental methods and results have been inconsistent. For instance, when musicians wearing EEG caps were asked to perform a familiar musical piece and enter flow, resting state data recorded after the performance showed higher power in upper alpha and beta in the frontal region of the brain, while functional connectivity, specifically phase slope index, in theta increased in frontal and temporal/parietal regions (Tan et al., 2024). In a separate study, participants completed a mental arithmetic task while wearing EEG caps (Katahira et al., 2018). Theta power increased during more challenging tasks and tasks that adapted to participants’ skill levels, compared to tasks that were overly easy or boring. Tasks that adapted to participants’ skills were classified by the authors as the “flow condition”. Because multiple frequency bands show increased power in the frontal cortex during flow, it is plausible that attention is regulated through diverse neural mechanisms, as suggested by Weber et al. (2009). Therefore, it is critical to study flow using tasks that both engage these broad neural processes and allow for their measurement during movement.

Dance may be an exceptional method to examine flow because it is a movement arts practice that requires heightened attentional awareness during engagement in highly challenging skills often in performative settings. Research has shown that dancers commonly experience high levels of flow in their daily lives (Hefferon & Ollis, 2006; Jaque et al., 2020; Thomson & Jaque, 2012, 2016). Dancers tend to have autotelic personalities known as “flow personalities”, which refer to a personality type that desires challenge, demonstrates excellent concentration skills, and is curious and intrinsically motivated (Hefferon & Ollis, 2006; Jaque et al., 2020; Thomson & Jaque, 2012, 2016). Autotelic personality types tend to experience high levels of dispositional flow (Baumann, 2021). Previous studies on flow have focused on classical and eurocentric dance forms (Hefferon & Ollis, 2006; Thomson & Jaque, 2012). Although street dance forms have gained global recognition through national and international competitions, most notably with Breaking’s inclusion in the 2024 Summer Olympics, they remain significantly understudied in academic and clinical research. Thus, there is a need to understand behavioral and neural interplay during street dance forms and how this relationship impacts the health and performance of dancers.

Memphis Jookin’ is a unique dance style that originated in Memphis, Tennessee, United States in the 1980s, and is one dance form that epitomizes flow. Originally termed “Gangsta Walking”, the style started off as a “walk with a bounce” to the “gangsta rap” music produced by local Memphis artists. The style has evolved over the years and gained worldwide popularity as an exquisite art form with elegant steps, slides, glides, toes spins, and footwork. Marico “Dr. Rico” Flake, one of the primary influencers of Memphis Jookin’ notes: *“Gangsta expression is an essence of an unapologetic attitude and is signified by a mindset that is both assertive and aggressive. In Memphis Jookin, it is exhibited through masculine movements and stops with the body reflected as speech while the feet are used as the mouth to communicate the message”* (Moore & Flake, n.d.)

The goal of our study was to examine how trait and state elements of flow are experienced during the performance of Memphis Jookin’ by professional dancers. Our first aim was to investigate the relationship between brain state and self-reports of emotional/mental states during improvisational and choreographed movement sequences. Our second aim was to look at the association between flow and other emotional/mental states during these movement experiences. We hypothesized that professional dancers of Memphis Jookin’ would report heightened states of flow during choreographed and improvised movement sequences. Additionally, we hypothesized that these professional dancers would achieve higher states of flow in comparison to lay people participating in other movement experiences (e.g., running, weight lifting, yoga). At the neural level, we hypothesized that heightened flow would be accompanied by (1) increased upper alpha activity (10–12 Hz) in the frontal lobe, reflecting attentional processes, and (2) enhanced theta-band functional connectivity (4–8 Hz) across the temporal and parietal lobes, consistent with Tan et al.’s (2024) findings in musicians. We also hypothesized that dance would be associated with theta activity, particularly in the premotor and motor cortices, given its relevance in spatial navigation (Chrastil et al., 2022; Do et al., 2021) and association with challenging/stimulating tasks (Katahira et al., 2018).

Our results supported our hypotheses. Alpha oscillations, associated with internal attention, were observed while watching dance and dancing to choreographed pieces, but showed less power during improvised dance. Active dance (choreographed and improvised) was associated with higher power in theta in comparison to watching dance and resting state. These novel findings regarding theta oscillations highlight the neural correlates of flow and spatial navigation in the performing arts and can inform future studies examining the impact of diverse dance forms in clinical populations.

## Results

### Demographics

All participants (n=6) were members of *Memphis Jookin’: The Show* and self-identified as professional dancers living in an urban community with an extensive history of dance training (**Supplementary Table 1**; self-reported from 10 to 20 years). Participants were primarily male (n=5, female: n=1), Black/African American (n=5, White/Causian: n=1), and Non-Hispanic (n=6) (Table 2). Participants’ ages ranged from 16–33, with an average age of 24.5.

### Behavioral Outcomes

#### Dispositional Flow and Flow State

##### Dispositional Flow:

Most dancers scored near maximum (maximal value on scale is 5.00) on the Short Dispositional Flow Scale (M = 4.81, SD = 0.27) and the Core Dispositional Flow Scale (M = 4.92, SD = 0.13) (Figure 2). Compared with published reference samples (dispositional flow during physical activity: *M* = 3.82, *SD* = 0.49, *N* = 616; core dispositional flow during extracurricular activity: *M* = 4.41, *SD* = 0.59, *N* = 2202), dancers scored significantly higher on dispositional flow (t=8.91, p<0.001, d=2.04) and core dispositional flow (t=9.10, p<0.001, d=0.86), both with a large effect size (Figure 2) (S. Jackson et al., 2010).

##### Flow State:

Similar to dispositional flow, dancers reported experiencing almost maximal flow (maximal value on scale is 5.00) during their performance of Memphis Jookin’: The Show through the Short Flow State Scale (Mean: 4.81, SD: 0.23) and Core Flow State Scale (Mean: 4.98, SD: 0.04) (Figure 2). Compared with published reference samples (flow state during physical activity: *M* = 3.77, *SD* = 0.56, *N* = 605; core flow state during sport: *M* = 3.37, *SD* = 0.74, *N* = 220), dancers scored significantly higher on flow state (t=10.84, p<0.001, d=1.87) and core flow state (t=30.67, p<0.001, d=2.2) in response to an acute dance experience, both with a large effect size (Figure 2) (S. Jackson et al., 2010). Additional qualitative reports of dance-induced flow can be found in **Supplementary Table 2**.

#### Trait Elements of Dance

Based on Bonferroni correction, an alpha value of 0.006 was used to evaluate statistical significance. With regard to interoceptive awareness, participants reported high levels of attention regulation (M = 4.76, SD = 0.28), body listening (M = 4.50, SD = 1.07), emotional awareness (M = 4.77, SD = 0.29), noticing (M = 4.58, SD = 0.68), not-worrying (M = 3.89, SD = 0.91), self-regulation (M = 4.75, SD = 0.32), and trusting (median: 5.00, IQR: 0.00) (Figure 3). Reports of attention regulation (*t* = 8.10, *p* < 0.001, *d* = 1.53), emotional awareness (*t* = 4.84, *p* = 0.003, *d* = 0.95), self-regulation (*t* = 6.57, *p* < 0.001, *d* = 1.21), and trusting (*t* = 21.19, *p* < 0.001, *d* = 1.18) were significantly higher, each with large effect sizes, than those reported by students and instructors trained in mind-body techniques, indicating elevated interoceptive awareness in our experienced dancers (Figure 3) (Mehling et al., 2012). Body listening (t=2.28, p=0.07, d=1.14), noticing (t=2.29, p=0.07, d=1.09), and not-worrying (t=1.65, p=0.16, d=0.74) showed no significant difference to this comparator group. Interestingly, reports of not-distracting (M = 2.06, SD = 0.68) were significantly lower than those reported by the mind-body techniques students and instructors in Mehling et al. (2012) (t=−4.06, p=0.009, d=1.32) (Figure 3).

#### State Elements of Dance

Based on Bonferroni correction, an alpha value of 0.01 was used to evaluate statistical significance. For the Multidimensional Impacts of Movement Scale, dancers scored in maximal ranges (maximal value on scale is 45.00) regarding the impact of dance on the body (M = 42.33, SD = 3.08), energy (M = 42.67, SD = 2.25), mind (M = 43.33, SD = 3.14), intuition (M = 42.83, SD = 2.40), and contentment (M = 44.33, SD = 1.63) (Figure 4). Body scores were significantly higher than those whose primary movement practice was running (t=5.73, p<0.001, d=2.95), but not yoga (p>0.01) or weightlifting (p>0.01). Energy scores were significantly higher in dancers than yogis (t=6.10, p<0.001, d=2.12), runners (t=8.83, p<0.001, d=3.51), or weight lifters (t=6.44, p<0.001, d=1.93). Mind scores were significantly higher in dancers than yogis (t=5.07, p=0.002, d=2.22), runners (t=7.47, p=0.002, d=3.8), or weight lifters (t=5.77, p<0.001, d=2.22). Intuition scores were significantly higher in dancers than yogis (t=6.33, p<0.001, d=2.31), runners (t=9.65, p<0.001, d=4.19), or weight lifters (t=6.53, p<0.001, d=2.21). Contentment scores were significantly higher in dancers than yogis (t=9.57, p<0.001, d=2.72), runners (t=11.82, p<0.001, d=3.58), or weight lifters (t=9.84, p<0.001, d=2.44). All effect sizes were large. Total MIMS scores were also significantly higher in dancers than yogis (t=7.01, p<0.001, d=7.26), runners (t=10.632, p<0.001, d=10.02), or weight lifters (t=7.66, p<0.001, d=7.7), paired with large effect sizes.

#### Relationships between Behavioral Outcomes

We examined the association between short dispositional flow and two of our other behavioral outcomes: interoceptive awareness and the multidimensional impact of movement. Short dispositional flow was chosen because all reports of flow were similarly high, dispositional flow assesses the general impact of dance, and short dispositional flow had a wider distribution than core dispositional flow. Because of the study’s low sample size, the goal of this analysis was to determine if there are strong correlations that warrant further investigation with a larger sample of dancers.

Dispositional flow was positively correlated with two aspects of interoceptive awareness: attention regulation (p = 0.005) and emotional awareness (p = 0.005) (**Supplementary Table 3**). Dispositional flow was trending towards significance in its correlation with dance’s impact on energy (p = 0.057) (**Supplementary Table 3**).

### Power spectral density metrics at the level of components

#### Overall assessment of PSDs:

Permutation-based analyses revealed frequency-specific modulations in EEG spectral peaks across behavioral conditions. Omnibus permutation tests showed significant main effects of condition for both the proportion of components exhibiting peaks and the total peak power, particularly within the theta (χ^2^ = 22.34, *p* < 0.001) and alpha (χ^2^ = 9.07, *p* = 0.005) bands. For total peak power, F-like permutation tests similarly indicated robust between-condition differences in the theta (F = 0.64, *p* < 0.001) and alpha (F = 0.70, *p* < 0.001) bands, with a trend observed in beta (F = 8.52, *p* = 0.051).

Planned pairwise contrasts revealed that alpha-band total peak power was significantly greater during pre- and post-baseline compared to choreography (*p* = 0.014, Δ = +211.7% and +306.1%, respectively) and improvisation (*p* = 0.014, Δ = +671.4% and +905.0%, respectively). Alpha power also trended toward being higher during watching dance than during choreography (*p* = 0.062, Δ = +277.9%) and improvisation (*p* = 0.062, Δ = +835.0%).

In contrast, theta-band total peak power was elevated during choreography and improvisation relative to pre-baseline (choreography: *p* = 0.062 [trend], Δ = +289.8%; improvisation: *p* = 0.032, Δ = +349.6%), post-baseline (choreography: *p* = 0.047, Δ = +440.9%; improvisation: *p* = 0.032, Δ = +514.8%), and watching dance (both *p* = 0.014, ≈ +100% relative increase). Together, these findings demonstrate a shift from alpha-dominated activity during rest and observation to heightened theta oscillations during creative movement. The following sections detail PSD findings for each identified component, along with descriptive characterizations of their spectral profiles.

#### Right Posterior Cingulate Gyrus:

Near the right posterior cingulate gyrus of Participant 1, we found notable peaks across all frequency bands. Theta was observed during dance experiences (Choreographed Dance: n_peaks_ = 1, min/max = 5.31 dB; Improvised Dance n_peaks_ = 3, min = 5.08 dB, max = 7.87 dB), but not during resting state nor watching dance (Figure 5). Alpha power was highest during pre-baseline (n_peaks_ = 1, min/max = 13.1 dB) and post-baseline (n_peaks_ = 2, min = 8.19 dB, max = 14.0 dB) (Figure 5). Watching dance was the only experience that drove activity in beta (n_peaks_ = 2, min = 2.15 dB, max = 3.98 dB) (Figure 6). Gamma power was also high during resting state, with a single peak observed for pre-baseline (6.16 dB) and post-baseline (4.37 dB) (Figure 5).

#### Left Middle Occipital Gyrus:

Similar to what we observed in the right posterior cingulate gyrus for Participant 1, theta activity was also present as a single peak for choreographed dance (power: 2.75 dB) and improvised dance (power: 2.99 dB) in the left middle occipital gyrus (Figure 6). Alpha was also present during these movement experiences (Choreographed Dance: n_peaks_ = 2, min 1.88 dB, max = 1.99 dB; Improvised Dance n_peaks_ = 2, min = 2.39 dB, max = 2.71 dB) where power was similar to that observed while watching dance (n_peaks_ = 4, min = 2.38 dB, max = 3.76 dB) (Figure 6).

Similar trends were seen in other areas and specific results from the left inferior temporal gyrus (**Supplementary Figure 1**), right paracentral lobule (**Supplementary Figure 2**), right supplementary motor area (**Supplementary Figure 3**), left posterior cingulate gyrus (**Supplementary Figure 4**), and right Rolandic operculum (**Supplementary Figure 5**) can be found in **Supplementary Information**.

### Functional Connectivity Metrics

#### Theta Connectivity:

Regarding theta, functional connectivity increased from pre- to post-dance in all three region types. In the left hemisphere (n_pre_ = n_post_ = 561 edges), the mean connectivity rose from *M*_pre_ = 0.075 to *M*_post_ = 0.083, a mean change of Δ*M* = 0.008 (95% CI [0.005, 0.010]) (Figure 7A). The effect size was small to moderate (Cohen’s *d* = 0.375), and the permutation test was significant (*p*_perm_ < .001), remaining significant after Holm correction across all band × region tests (*p*_holm_ = .005). The right hemisphere showed a highly similar pattern (n = 561), albeit with a much larger effect size, with *M*_pre_ = 0.066 and *M*_post_ = 0.096 (Δ*M* = 0.030, 95% CI [0.027, 0.033], *d* = 1.114; *p*_perm_ < .001; *p*_holm_ = .005) (Figure 7B). The interhemispheric network (n = 1,156) also increased with a large effect size: *M*_pre_ = 0.066 to *M*_post_ = 0.087 (Δ*M* = 0.022, 95% CI [0.020, 0.023], *d* = 1.232; *p*_perm_ < .001; *p*_holm_ = .005) (Figure 7C). Anatomically speaking, the observed increases were attributable to amplified intra-prefrontal/frontal coherence together with increased prefrontal/frontal–temporal coupling.

#### Alpha Connectivity:

Alpha functional connectivity also increased in the left hemisphere (n = 561), *M*_pre_ = 0.121 to *M*_post_ = 0.141 (Δ*M* = 0.020, 95% CI [0.015, 0.025], *d* = 0.500; *p*_perm_ < .001; *p*_holm_ = 0.005) (Figure 8A) and interhemispherically (n = 1,156), *M*_pre_ = 0.132 to *M*_post_ = 0.141 (Δ*M* = 0.009, 95% CI [0.006, 0.013], *d* = 0.236; *p*_perm_ < .001; *p*_holm_ = 0.005) (Figure 8C). Though an increase was seen in the right hemisphere (n=561), the increase was non-significant, *M*_pre_ = 0.137 to *M*_post_ = 0.141 (Δ*M* = 0.004, 95% CI [0.001, 0.009], *d* = 0.086; *p*_perm_ = 0.149; *p*_holm_ = 0.149) (Figure 8B). Anatomically speaking, the observed increases were attributable to amplified intra-prefrontal/frontal coherence together with increased prefrontal/frontal–cingulate coupling.

Results for beta (**Supplementary Figure 6**) and gamma connectivity (**Supplementary Figure 7**) can be found in the **Supplementary Information**.

## Discussion

Due to previous technical limitations in neuroimaging, the dynamic brain in motion has remained largely understudied. However, recent advances in mobile brain imaging have enabled us to investigate the neural signatures of movement (King & Parada, 2021; Yen et al., 2023). Among professional Memphis Jookin’ dancers (n=6), we characterized trait and state dimensions of dance and quantified neurophysiological differences between rest and choreographed/improvised sequences in 1 dancer. In comparison to other physical activity practices, dance induced heightened states of flow, interoceptive awareness, and mind-body integration. Excitingly, compared to the brain at rest, dance induced heightened levels of theta activity in the right and left posterior cingulate gyri, left inferior temporal gyrus, left middle occipital gyrus, right paracentral lobule, right supplementary motor area, and right Rolandic operculum, regions supporting sensorimotor, visual, and self-referential domains. This marks the first evidence of theta oscillatory activity during dance movement in the human brain, suggesting that theta rhythms may serve as a neural signature of the integration between motor intention, sensory processing, and emotional engagement, fundamental components of performative dance and the flow state. Additionally, we found evidence of increased within-hemisphere functional connectivity in key brain regions associated with self-referential processing, emotion regulation, and social awareness.

Dancers reported high levels of both state and trait flow, significantly exceeding standardized scores typically observed in sport, physical, and other extracurricular activities. These quantitative findings were reinforced by qualitative accounts, such as one dancer’s reflection: ‘I am fully immersed in what I am doing. The experience is very focused.’ These findings build on prior work demonstrating dance’s capacity to elicit flow states, immersive experiences characterized by full presence, heightened satisfaction, optimal performance, and strengthened embodied awareness (Gang & Zhang, 2023). Unlike many other physical activities, dance integrates movement with music/rhythm, emotion, and social connection (Basso et al., 2021), making it uniquely conducive to flow. Importantly, research indicates that dance-induced flow supports not only artistic cultivation and self-realization but also reduces stress, improves mood and self-confidence, and fosters creativity, underscoring its value for both performance and personal flourishing (Gang & Zhang, 2023). Importantly, subjective flow during dance has been linked to distinct autonomic patterns, with heightened flow associated with a co-inhibited autonomic nervous system profile (Jaque et al., 2020). This co-inhibited profile reflects a flexible balance between sympathetic and parasympathetic activity, the very capacity cultivated through many mind–body-movement practices, including yoga and dance, which train the ability to shift fluidly between states of activation and relaxation.

Additionally, dancers demonstrated heightened levels of trait interoceptive awareness compared to individuals trained in mind-body practices such as meditation/mindfulness, yoga, Tai Chi, Feldenkraïs method, Alexander technique, breath therapy, massage, or body-oriented psychotherapy. In particular, dancers showed heightened scores in attention regulation, emotional awareness, self-regulation, and trusting, suggesting that dance strengthens the capacity to attend to bodily sensations, integrate them with emotions, and relate to the body as a reliable resource for regulation and well-being. Such skills are central to artistry and performance, where the capacity to channel emotion through the body, maintain composure under pressure, and trust embodied intuition distinguishes dance as a deeply integrative practice (Houston, 2024; Long, 2024). Interestingly, dancers showed lower scores on the not-distracting subscale, suggesting they are more likely to block out sensations of pain and discomfort. This pattern is consistent with the culture of dance, where physical discomfort is common yet often overridden by the expectation that ‘the show must go on.’ Prior research indicates that while this capacity may enhance resilience and performance focus, it can also contribute to higher injury risk by encouraging dancers to ignore bodily signals that would otherwise prompt rest or recovery (Soundy & Lim, 2023). These findings highlight the dual nature of attentional control in dance: adaptive for artistic expression, but potentially maladaptive for long-term health. Overall, these findings support other literature demonstrating that dancers, compared to control participants, show enhanced interoceptive accuracy in a heart beat perception task (Christensen et al., 2018). Additionally, evidence for causal effects comes from a nonrandomized controlled trial in community-dwelling older adults, which demonstrated increased interoceptive awareness after 12-weeks of a psychomotor intervention mediated by creative dance (Rosado et al., 2025). These findings regarding the impact of dance on interoceptive awareness have important implications for aging populations, in whom this critical capacity is often diminished leading to falls and injury (Murphy et al., 2018).

Dancers also demonstrated consistently high scores on the MIMS, reflecting strong trait bodily awareness, heightened energy, positive psychological well-being, trust in their own thoughts and emotions, and overall life satisfaction. Except for the body subscale, these scores were significantly higher when compared to yogis, runners, or weightlifters suggesting that dance may be superior to other forms of physical activity at improving psychological health. This is the first time that MIMS has been used to assess the body-mind state of dancers; however, these findings align with prior work demonstrating that dance is superior to other forms of physical activity at reducing distress and enhancing motivation, memory, and social cognition (Fong Yan et al., 2024). Taken together, these converging findings support the notion that dance provides unique benefits for mind–body integration, extending beyond the effects typically observed with other forms of physical activity.

Furthermore, dispositional flow was positively associated with the MAIA subscales of attention regulation and emotional awareness, suggesting that flow supports dancers’ ability to sustain attention to bodily sensations and recognize their link to emotional states. These capacities are particularly critical in performative contexts, where precise regulation of both physical and mental states is essential for optimal performance (Martín-Rodríguez et al., 2024; Swann et al., 2017).

During both choreographed and improvisational dance, theta activity in the 4 to 8 Hz range was found in several brain regions including the right and left posterior cingulate gyri, left inferior temporal gyrus, left middle occipital gyrus, right paracentral lobule, right supplementary motor area, and right Rolandic operculum. This is the first time that theta activity has been seen in the human brain during dance.

Extant literature in rodents has identified that theta activity is invariably present in the hippocampus during physical movement, including during spatial navigation, wheel, and treadmill running, with this activity proportionally increasing with running speed. During theta, the hippocampus is bathed in acetylcholine which is thought to facilitate plasticity by biasing the hippocampus towards receiving afferent input from the entorhinal cortex rather than recurrent input from CA3 (Buzsáki, 2005; Giocomo & Hasselmo, 2007). This enables the hippocampus to receive and learn new information about the environment during spatial navigation. This information is then passed to the cortex, supporting hippocampal-cortical memory consolidation (Colgin, 2016). Studies using intra-hippocampal EEG recordings in walking humans have also shown theta activity present during movement (Bohbot et al., 2017), with theta activity increasing with increasing movement speeds (Aghajan et al., 2017). Additionally, a recent study comparing physical versus virtual navigation found that physical movement supported greater theta power as well as better memory performance (Maidenbaum et al., 2025). Few studies, however, have investigated neural oscillations during moving humans in real world, non-task based scenarios.

One investigated the neural correlates of movement in five butoh dancers (Theofanopoulou et al., 2024). Butoh is a Japanese dance form characterized by slow, controlled movements and depicting subjects such as aging, death, and other serious aspects of the human experience. Performers were hyperscanned during a live performance and resulting gamma (30–50 Hz) interbrain synchrony was calculated and visualized through artistic presentation. Additionally, a recent study in 40 dyads investigated EEG in combination with electrooculography, electromyography, and motion capture, and demonstrated the feasibility of examining event-related potentials during movement (Bigand et al., 2025). Using multivariate temporal response functions, they showed that acoustic events elicited the P50–N100–P200 complex, movement initiation elicited central lateralized movement-related cortical potentials, and movement observation elicited the occipital N170. The authors note that the occipital N170 is a novel marker of social engagement, which occurs specifically when observing others move. Our data add to the growing literature showing that observing dance in a social context engages brain activity that is distinct from baseline processes and also differs from the neural patterns associated with dancing itself. Additionally, studying the brain dynamics of a person experiencing flow during dance introduces an interesting interplay between flow and spatial navigation. During performance, dancers often demonstrate heightened spatial awareness and must be cognizant of the effects of their movements on their location in space. Previous work has demonstrated that humans show heightened theta activity during active spatial navigation tasks assessed through mobile EEG (Chrastil et al., 2022; Do et al., 2021). Here, we newly demonstrate that dancers also experience heightened theta power during active performance.

In comparison to pre-dance resting measures, all four frequency bands (theta, alpha, beta, and gamma) showed significant post-intervention increases in connectivity across left, right, and interhemispheric networks, even after controlling for the familywise error rate. The only decrease we found was in beta activity in the right hemisphere. These results indicate a broad strengthening of functional coupling that is pronounced both within- and between-hemisphere networks. These effects were particularly strong within and between prefrontal/frontal and temporal cortices.

Using resting-state fMRI, prior work found stronger functional connectivity in dancers than in non-dancers across the putamen, globus pallidus, posterior cerebellum, and anterior insula, suggesting tighter integration between metacognitive processes (e.g., the conscious understanding of one’s own physical and mental processes) and motor, spatial, rhythmic, and emotional systems. The authors suggest, therefore, that this increase in functional connectivity may serve as a neural mechanism of embodied cognition (Yang et al., 2024). Another study comparing dancers to non-dance controls revealed increased functional connectivity in the precentral gyri, postcentral gyri, and bilateral putamen as well as between the middle cingulate cortex and bilateral putamen and precentral and postcentral gyri, indicating enhanced functional connectivity in the cortic-basal ganglia loops (Li et al., 2015). Finally, another key study in the field integrating diffusion tensor, morphometric, and resting state and task-related fMRI, found different patterns of functional connectivity in dancers versus non-dancers in both the action observation network (thalamus and frontotemporal pole) as well as a broadly defined motor circuit (e.g., between superior parietal lobule and precentral and postcentral gyrus; between primary motor cortex and fusiform cortex) (Burzynska et al., 2017). Interestingly, this increase in functional connectivity was associated with an objective measure of dance skill (i.e., performance on Dance Central video game) (Burzynska et al., 2017). Our results add to this literature indicating that dance can acutely increase functional connectivity in similar regions involved with self-referential processing, emotion regulation, and social awareness. Future studies are needed to examine the relationship between acute changes in functional connectivity and changes in dance-induced mental states.

Overall, these findings suggest that dance provides a strengthening of coordinated activity, especially within-hemisphere coordination, plausibly reflecting tuning of cortico-cortical circuits that subserve domain-specific processing (e.g., sensorimotor and associative networks) while leaving cross-callosal integration comparatively stable. In practical terms, this pattern is compatible with more efficient local processing modules that may underpin improved performance on tasks supported by those networks. These neural findings align with the behavioral findings of superior sensorimotor integration and motor control seen in dancers (Bläsing et al., 2012; Burzynska et al., 2017).

Some limitations of this project include the small sample size and the limited diversity in dance experience compared to larger and more varied control groups. These constraints highlight the need for future studies with larger, more heterogeneous cohorts of dancers. Longitudinal designs would also be valuable to assess the causal relationship between dance practice and heightened reports of flow and interoception. Additionally, primary means of assessing flow state and other psychological variables were self-report. Future studies could incorporate cognitive tasks that focus on creativity such as the Remote Associates Task or the Alternate Uses Task. Integration of EEG with other physiological measures such as heart rate variability or electrodermal activity can capture aspects of the peripheral nervous system. Additionally, comparison of other professional sports activities such as capoeira, basketball, or other team sports can provide more insight on flow and its associated brain dynamics across different forms of physical activity. Finally, there is great potential in examining the impact of dance in individuals with known interoceptive or emotion-regulation difficulties (e.g., depression, anxiety, attention-deficit/hyperactivity disorder, autism spectrum disorder) to evaluate therapeutic applications.

In conclusion, dance elicited convergent behavioral and neurophysiological change: self-reported flow, interoceptive awareness, and mind–body integration rose in step with increases in theta-band activity and large-scale functional connectivity. Using mobile EEG, we captured these effects in situ during real-world dance, establishing ecological validity that is rarely achievable in laboratory paradigms. The alignment between subjective experience and neural dynamics frames dance not only as physical performance but as a cognitive–affective practice that reorganizes network coordination. Methodologically, we show that mobile EEG can track these dynamics outside the lab, enabling the study of socially embedded movement with high temporal resolution. This integrative framework points to practical applications in rehabilitation, mental health, and education, where embodied practices can be monitored and tailored in everyday settings.

## Methods

In Spring 2022, six Memphis Jookers underwent a series of experiments to examine the behavioral and brain manifestations of Memphis Jookin’. Both trait and state flow were assessed and examined in relation to interoceptive awareness and multidimensional impacts of movement. Because this was a case series, we utilized standardized data from other studies to compare the present results. Demographic and physical activity behaviors were captured, and qualitative assessments of flow were also examined. Additionally, we recorded brain activity through mobile EEG during the performance of both choreographed and improvisational movement. As an additional outcome, we sought to delineate the brain states associated with different types of dance (e.g., choreographed versus improvised). All participants gave their informed consent prior to study participation, including giving consent for publication of identifying images in an online open-access publication. The study was conducted in accordance with the relevant guidelines and regulation and this study protocol was approved by the Virginia Tech Institutional Review Board (VT IRB 22–146).

### Self-Report Questionnaires

Participants were instructed to complete a series of self-reported questionnaires in regard to 1) their daily habits; 2) their general disposition and dance experience; or 3) their performance of Memphis Jookin’: The Show, which occurred the previous evening.

#### Assessments on flow associated with general dance and specified performance:

##### Short Dispositional Flow Scale-2 (S. A. Jackson et al., 2008):

The Short Dispositional Flow Scale consists of 9 items, with one item from each of the four-item measures of the nine flow dimensions presented in the Dispositional Flow Scale 2 (S. A. Jackson & Eklund, 2002) that measure the participant’s general tendency to experience flow. Participants were asked about flow in relation to their general dance experience. Responses were rated on a five-point Likert scale from 1 (“Never”) to 5 (“Always”). This scale provides a valid and reliable assessment of the autotelic personality (e.g., “I am completely focused on the task at hand”).

##### Core Dispositional Flow Scale (Martin & Jackson, 2008):

The Core Dispositional Flow Scale is a 10-item scale aimed to measure the subjective optimal experience of flow itself. Participants were asked about their experience of flow in relation to their general dance experience. Responses were rated on a five-point Likert scale from 1 (“Never”) to 5 (“Always”). This scale provides a valid and reliable assessment of the central, or core, subjective experience of being in flow (e.g., “I am in the zone”).

##### Short Flow State Scale-2 (S. A. Jackson et al., 2008):

The Short Flow State Scale is a 9-item scale (with one item from each of the four-item measures of the nine flow dimensions presented in the Flow State Scale 2 (S. A. Jackson & Eklund, 2002) that aimed to capture aggregate or global flow during the participant’s performance of Memphis Jookin’: The Show. Questions were the same as in the Short Dispositional Flow Scale but were asked in the past tense in relation to the previous night’s performance (e.g., “I was completely focused on the task at hand”). Responses were rated on a five-point Likert scale from 1 (“Never”) to 5 (“Always”).

##### Core Flow State Scale (Martin & Jackson, 2008):

The Core Flow State Scale is a 10-item scale that aims to measure the subjective experience of flow during the participant’s performance of Memphis Jookin’: The Show. Questions were the same as in the Core Dispositional Flow State Scale but asked in the past tense in relation to the previous night’s performance (e.g., “I was in the zone”). Responses were rated on a five-point Likert scale from 1 (“Never”) to 5 (“Always”). This scale provides a valid and reliable assessment of the central, or core, subjective experience of being in flow.

#### Trait Elements of Dance:

##### Multidimensional Assessment of Interoceptive Awareness (MAIA) (Mehling et al., 2012):

The MAIA was used to evaluate a participant’s level of interoceptive awareness as it pertains to their daily life. The MAIA is a valid and reliable tool, which consists of an eight-scale state-trait questionnaire with 32 items that measures multiple dimensions of interoception. Subscales included noticing, not-distracting, not-worrying, attention regulation, emotional awareness, self-regulation, body listening, and trusting, which are related, but independent, dimensions of interoceptive attention, appraisal, and self-regulation. Participants were asked to indicate how often each statement applies to them in daily life. Responses were rated on a range from “Never” (0) to “Always” (5), with specific items for not-distracting and not-worrying reverse-scored. In a group of healthy adults, the MAIA subscales showed adequate goodness-of-fit indices with internal-consistency reliabilities ranging from 0.66 to 0.87, with unstandardized alphas over 0.70 for five of the eight scales (Mehling et al., 2012).

#### Assessments in relation to the performance of Memphis Jookin’: The Show:

##### Multidimensional Impacts of Movement Scale (MIMS) (Lynn & Basso, 2023):

MIMS was used to assess the multidimensional impacts of the participant’s movement practice as it pertained to the performance of Memphis Jookin’: The Show. MIMS is a 45-item scale developed based on the 5 Koshas, metaphorical layers that comprise the human body and mind. The Koshas stem from the Upanishads, the ancient Vedic texts that informed Hinduism and many aspects of yogic philosophy. The Koshas consist of the bliss body, intellectual body, emotional body, breath body, and physical body. MIMS explores how intentional movement practices can impact multidimensional aspects of an individual and their mind-body connection. There are 9 questions per Kosha, scored on a Likert scale from “Disagree” to “Agree”, with five subscales (i.e., body, energy, mind, intuition, contentment) and an overall MIMS score. In a group of yoga practitioners, runners, and weight lifters, test–retest reliability demonstrated stability over time (*r* = 0.737, *p* < 0.001), with Cronbach’s alpha between 0.775 and 0.840 for each of the factors, *p* < 0.001 (Lynn & Basso, 2023).

#### Descriptive Flow Experience

The descriptive flow experience was assessed based on two qualitative questions that asked for further insight into aspects of flow state. The first question inquired about the participant’s experience of flow while they were engaged in movement improvisation, while the second question asked about the experience of flow while they were engaged in choreographed movement sequences. We defined flow as a feeling of being deeply and fully immersed in the creative experience and able to experience peak performance. Neither of these questions were previously validated.

### Task description

**Figure 1:**
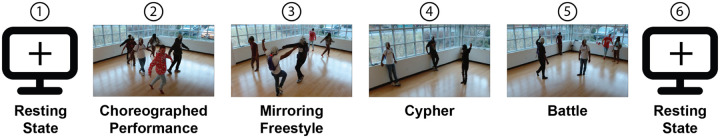
Overview of Behavioral Tasks. After 6 dancers of Memphis Jookin’ The Show completed validated assessments on flow and other psychological measures, 2 dancers wore 32-channel mobile EEG caps (LiveAmp 32, Brain Products GmbH, Gilching, Germany) and had their brain activity recorded while engaging in the following tasks: 1) Resting State Before Dance, 2) Choreographed Performance, 3) Mirroring Freestyle, 4) Cypher, 5) Battle, 6) Resting State After Dance.

**Figure 2:**
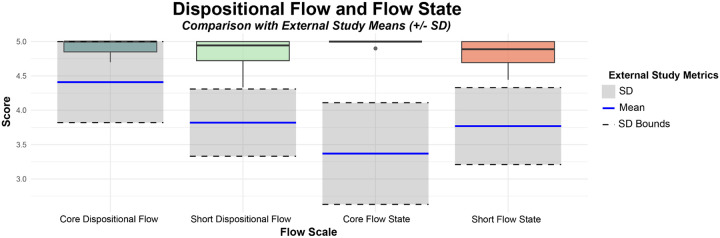
Reports of Core/Short Dispositional Flow and Flow State. Participants (N=6) reported experiencing high levels of flow during their general dance experience (Dispositional Flow) and during their performance of Memphis Jookin’: The Show (Flow State) the night before. Their scores were compared to short flow and core flow descriptive statistics collected from different studies reported in Jackson et al. (2010), p. 47.

**Figure 3:**
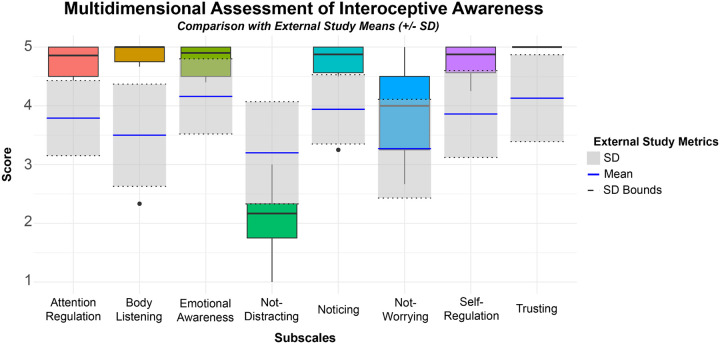
Interoceptive Awareness. Participants (N=6) reports of interoceptive body awareness were split into the 8 subscales of the Multidimensional Assessment of Interoceptive Awareness. Responses associated with Not-Distracting and Not-Worrying were reverse scored. Participants’ scores were compared to the means and standard deviations (SD) of a population (N = 325) of students and instructors experienced in body awareness techniques that included: meditation/mindfulness, yoga, Tai Chi, Feldenkraïs method, Alexander technique, breath therapy, massage, or body-oriented psychotherapy (Mehling et al., 2012).

**Figure 4:**
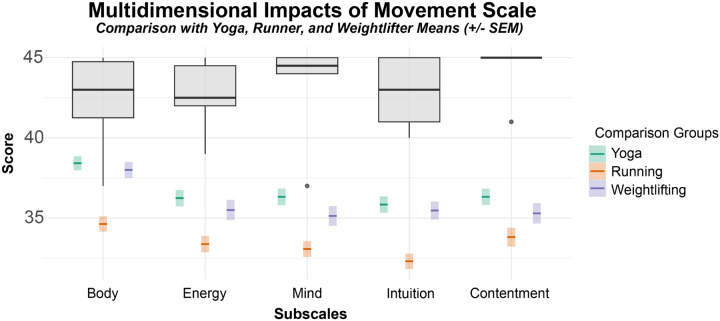
Multidimensional Impacts of Dance as a Movement. Participants (N = 6) reported a high impact of dance on their body, energy, mind, intuition and contentment. Participants’ scores were compared to the means and standard deviations (SD) of a population of individuals (n=103) whose primary movement form was yoga, running, or weightlifting (Lynn & Basso, 2023).

**Figure 5:**
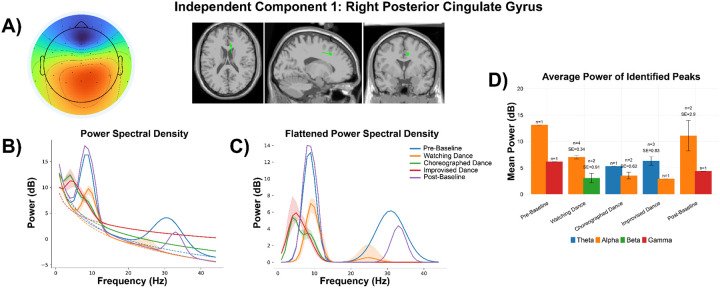
Participant 1 - Independent Component 1. **A)** Topographical distribution, with a classification of 99.9% brain according to ICLabel. Dipole modeled for the component through DIPFIT, with MNI coordinates of [8.0, 21.4, 37.9], a residual variance of 0.88%, and localization to the Right Posterior Cingulate Gyrus on the AAL3 atlas. **B)** Power spectral density modeled through specparam for each experience, including average aperiodic fit (dotted line) and average identified gaussian peaks (solid line). **C)** The aperiodic fits were removed from each modeled power spectra, providing a flattened spectra with only visible peaks. Shaded areas represent a full range of identified peaks (or lack thereof) for plots B and C. **D)** Average flattened power of identified peaks from the power spectra in Plot C split by experience and frequency band (Theta [4–8 Hz], Alpha [8–12 Hz], Beta [12–30 Hz], Gamma [30–45 Hz]). Number of peaks and standard error (SE) are shown above each bar. Error bars are +/− 1 standard error from the mean.

**Figure 6:**
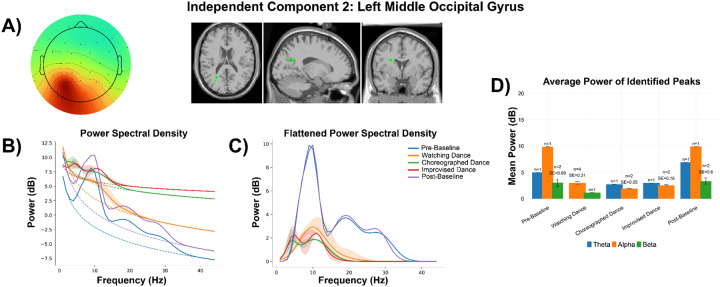
Participant 1 - Independent Component 2. **A)** Topographical distribution, with a classification of 98.0% brain according to ICLabel. Dipole modeled for the component through DIPFIT, with MNI coordinates of [−25.1, −58.9, −23.9], a residual variance of 1.76%, and localization to the Left Middle Occipital Gyrus on the AAL3 atlas. **B)** Power spectral density modeled through specparam for each experience, including average aperiodic fit (dotted line) and average identified gaussian peaks (solid line). **C)** The aperiodic fits were removed from each modeled power spectra, providing a flattened spectra with only visible peaks. Shaded areas represent a full range of identified peaks (or lack thereof) for plots B and C. **D)** Average flattened power of identified peaks from the power spectra in Plot C split by experience and frequency band (Theta [4–8 Hz], Alpha [8–12 Hz], Beta [12–30 Hz]. No Gamma peaks (30–45 Hz) were identified. Number of peaks and standard error (SE) are shown above each bar. Error bars are +/− 1 standard error from the mean.

**Figure 7:**
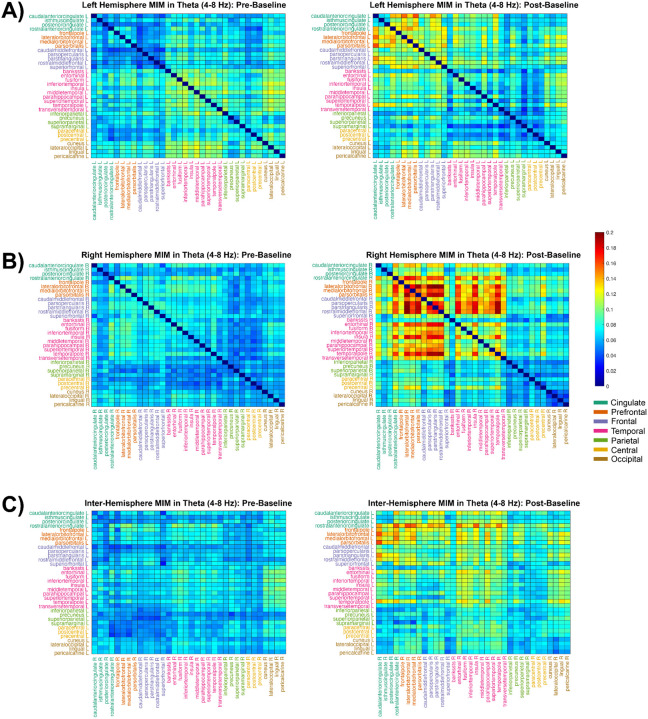
Functional Connectivity in Theta (4–8 Hz). Intra-hemispheric MIM calculations between 34 regions of interest in 7 cortical areas (defined by the Desikan-Killiany atlas) in the left (top) and right (middle) hemispheres during pre-baseline (left) and post-baseline (right). Inter-hemispheric MIM calculations are shown in the bottom row, where the Y-axis represents regions in the left hemisphere and the X-axis represents regions on the right hemisphere.

**Figure 8:**
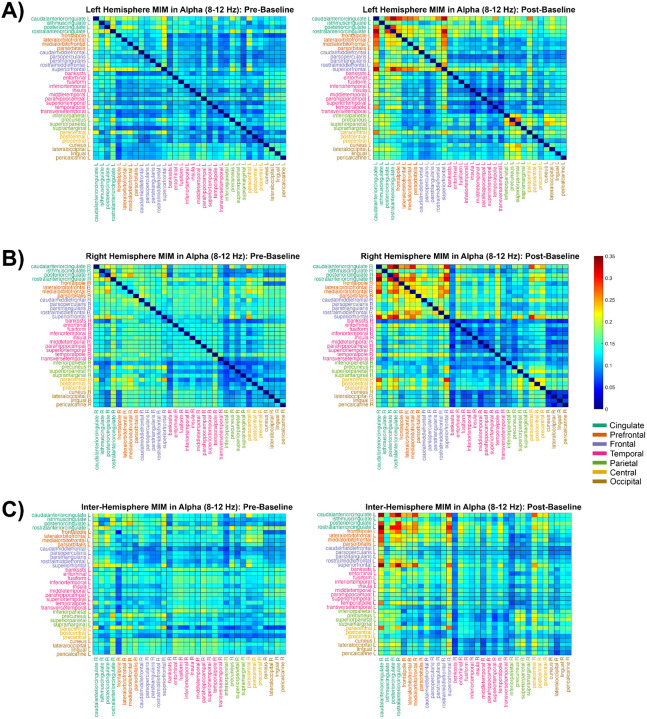
Functional Connectivity in Alpha (8–12 Hz). Intra-hemispheric MIM calculations between 34 regions of interest in 7 cortical areas (defined by the Desikan-Killiany atlas) in the left (top) and right (middle) hemispheres during pre-baseline (left) and post-baseline (right). Inter-hemispheric MIM calculations are shown in the bottom row, where the Y-axis represents regions in the left hemisphere and the X-axis represents regions on the right hemisphere.

The six dancers were asked to perform a series of choreographed and improvised dance sequences once they completed their psychological assessments (Figure 1). Two of the dancers who wore EEG caps first completed a resting state assessment where they were asked to stare at a crosshair on a computer for about 5 minutes (Pre-Baseline). Afterwards, all dancers performed two choreographed pieces from their previous night’s show (Choreographed Dance). Dancers then sectioned off into pairs and performed a series of improvised movements while their partner mimicked their movements (Mirroring Freestyle). Later, the dancers created a circle and performed improvised movements one-by-one while other dancers watched and responded with a series of their own movements (Cypher). Lastly, similar to the cypher, dancers were split into pairs again, but took turns with their improvised movements in response to one another’s movement sequences (Battle). Because our study was interested in the neural differences between choreographed and improvised dance, data collected during Mirroring Freestyle, Cypher, and Battle were averaged to represent improvised dance, unless otherwise noted (Table 2). Once participants had completed all movement experiences, the two dancers wearing the EEG caps were asked to complete a similar resting state assessment for 5 minutes (Post-Baseline).

### Electroencephalography Collection and Pre-Processing

Mobile EEG was utilized to define quantitative measures of brain state during choreographed and improvisational movement practices. Two dancers wore a mobile 32-channel wet electrode EEG cap (LiveAmp 32, Brain Products GmBH, Gilching, Germany) while performing a variety of dance experiences. To understand baseline brain state, we asked both dancers to stare at a crosshair on a computer for 5 minutes while EEG data was recorded. Data was collected at 500 Hz and impedance for all electrodes was between 0–25 kΩ before recording began. Data from each participant during the entire session was pre-processed as a single dataset using EEGLAB 2025.0.0 (Delorme & Makeig, 2004). First, electrodes were subjectively assessed by trained researchers for proper signal to noise ratio. As electrodes placed near the occipital lobe (O1, Oz, O2 on the 10–20 system) displayed significant noise during the movement sequences, they were removed as the first step of pre-processing. The remaining 29 channels were then filtered using a finite impulse response filter with a low cutoff of 1 Hz and high cutoff of 45 Hz. Faulty EEG channels (defined as flat for 5+ seconds, containing high frequency noise 4+ standard deviations from the data, and/or correlating with nearby EEG channels <0.8) were removed. Remaining segments of data that were 20+ standard deviations different from artifact-free segments were selected for interpolation through Artifact Subspace Reconstruction (ASR) through a 0.5s window principal component analysis (Chang et al., 2018). After ASR, if any of the original 29 channels were removed, the removed channels were interpolated through spherical interpolation and added back to the dataset. Afterwards, the data was full rank re-referenced to the common average reference.

### Independent Component Analysis and Dipole Fitting

Once the data was pre-processed, the signal was decomposed using Adaptive Mixture Independent Component Analysis (AMICA) using a single model and 2000 iterations (Hsu et al., 2018). The AMICA algorithm has its own built-in function to reject bad samples of data that may impede the decomposition. AMICA uses an iterative process to fit an estimated model onto the data, while determining which samples of data should be rejected based on their log-likelihood difference from the mean of the data in standard deviations. Once a rejection occurs, the model re-estimates the best fit for the data until the next rejection takes place. For our analysis, the rejection was conducted for 15 iterations using a rejection criterion of 3 standard deviations. The rejection interval was set to 1, meaning that an outlier rejection was performed for 15 continuous iterations.

To model the macroscopic brain dynamics taking place during our study, we used EEGLAB’s DIPFIT plugin to fit dipole models onto our identified independent components. To perform dipole fitting, we used the template boundary element model based on the Montreal Neurological Institute (MNI) template brain (Oostenveld et al., 2001). We used the following vector to align our electrode coordinates to the MNI template brain: [0.885926 −15.7509 0.726516 4.74214e-08 −7.41516e-08 −1.5708 1 1 1], where the first three values represent X,Y,Z translation (mm), the second three values represent X, Y, Z rotation (radians), and the last three values represent X, Y, Z scaling (a value of 1 means no scaling). To account for bilaterally symmetric dipoles, we used the fitTwoDipoles plugin to automatically detect these individual dipoles and instead, fit two position-symmetric dipoles (Piazza et al., 2016).

Once dipoles were fitted onto each independent component, the plugin produced a set of MNI coordinates for each dipole. To map each dipole onto an anatomical region of interest, we used the Automated Anatomical Labelling atlas 3 version 2 (Rolls et al., 2020). Using each dipole’s MNI coordinates, we found their corresponding brain region on the atlas. If an exact match was not found, regions within a 10mm radius were searched and the nearest region was listed in our findings.

Finally, we used a machine learning classifier to label the potential sources generating the independent components identified through AMICA using the ICLabel EEGLAB plugin (Pion-Tonachini et al., 2019). After labelling independent components through ICLabel, only those classified with at least 50% brain activity were kept for further analysis. Once the independent components were pruned, each participant’s dataset was split into individual datasets for each experience, maintaining their calculated independent component weights.

### Computing and parameterizing power spectral density (PSD)

As a last step to ensure we were evaluating proper dipole models, only components with residual values less than 15% were used for further analysis. This threshold for high quality dipoles has been previously justified (Artoni et al., 2014).

PSDs for the time-series data for each independent component activation were then calculated using the Welch method (MATLAB’s *pwelch* function: nfft = 500, overlap = 500/2 Hz, window = 500 Hz). The power spectrum was then parameterized using the specparam Python package (version 2.0.0rc3), which performs an iterative process of fitting gaussians on identified peaks in a log-power spectrum while modelling the aperiodic component of the spectrum using a Lorentzian function (Donoghue et al., 2020). Settings for the algorithm were: peak width limits: 2–8 Hz, maximum number of peaks: 6, minimum peak height: 0.2 dB, peak threshold: 2 SD, and aperiodic mode: fixed. The algorithm parameterized the power spectra between 1 and 44 Hz. Final values for power were multiplied by 10 to convert them to decibels (dB), or 10*log(uV^2^/Hz).

Once all the independent component activations’ power spectra were modeled through specparam, the model was further fine-tuned using models’ goodness of fit metrics: error and R^2^. Any models that had an error that was 1.5*IQR above the 75th percentile or had an R^2^ value of 1.5*IQR below the 25th percentile were removed before final analysis.

We used power spectra from Participants 1 and 2 to determine the best algorithm to identify oscillatory activity during dance. However, given the limited data available from Participant 2, we only present power spectra from Participant 1 in our results.

### Computing Functional Connectivity

Movement experiences often contaminate EEG data with motion artifacts. As a result, ICA decompositions often identify less brain activity and more muscle-driven noise. This contamination makes it difficult to reliably apply source localization techniques to EEG data, so we only conducted functional connectivity analysis on our resting state data: Pre-Baseline and Post-Baseline. Pre-Baseline and Post-Baseline datasets were appended and preprocessed as singular datasets for each participant. Using only the raw datasets from the resting state periods for both participants, we performed our preprocessing pipeline as highlighted above, with a few notable changes. The occipital electrodes were kept for these datasets because they were not contaminated with noise and would allow us to identify additional independent components. Also, when independent components were identified through AMICA, they were immediately labeled with ICLabel and removed according to their categorization (at least 90% muscle, eye, heart, line noise, or “other”). Datasets were then split into their respective Pre-Baseline and Post-Baseline experiences and run through the same dipole fitting processing described above.

For each dataset, a leadfield matrix was calculated using the Colin27_5003_Standard-10–5-Cap339 template head model included in EEGLAB. To calculate inter-regional functional connectivity (FC) at source level, we used the ROIconnect plugin on EEGLAB (Pellegrini et al., 2023). The sensor data was then downsampled to 100 Hz and received a source projection filter (linearly-constrained minimum variance beamformer). Power was calculated using the Welch method and summed across voxels of regions identified according to the Desikan-Kilianny atlas (Desikan et al., 2006). Principal component analysis was performed for each region’s time series data and identified the 3 strongest components for each region. As recommended by Pellegrini et al. (2023), inter-regional FC was estimated by calculating the multivariate interaction measure (MIM) using the 3 strongest components identified for each region. MIM was then averaged across frequency ranges of interest (Theta [4–8 Hz], Alpha [8–12 Hz], Beta [12–30 Hz], Gamma [30–35 Hz]) for comparison across frequency bands. To generate summary statistics, if a specific matrix was calculated for intra-hemispheric connectivity (left-left or right-right), we used MATLAB’s *triu* function to extract the upper triangular portion of the connectivity matrix, above the main diagonal, and then calculated averages and standard deviations of unique connectivity data points.

Overall, we were interested in examining differences in functional connectivity (FC) between the two time points to assess the role of dance and flow on intra-brain synchronization. We assessed intra- and inter-hemispheric FC across frequency bands during pre-baseline and post-baseline. We compared FC in different cortical regions (e.g., cingulate, prefrontal, frontal, temporal, parietal, central, and occipital). Calculations for MIM are sensitive to noise and require clean EEG signals. As a result, we present findings from Participant 1, whose signals were clear enough to conduct a proper FC analysis and can hopefully guide future studies on the impact of dance on FC.

### Statistics

To delineate demographic factors, including age, sex, race, and ethnicity, we utilized descriptive statistics. To contextualize our behavioral findings, we compared psychological assessment scores against published reference samples. For this purpose, mean statistics were compared. Group differences were tested using Welch’s unequal variance *t*-test, appropriate for small sample sizes and unequal variances between groups (Two-Sample t-Test Calculator (Welch’s t-Test), n.d.). To examine relationships between behavioral outcomes, a Spearman’s rank-order correlation (df = 4) was conducted between reports of short dispositional flow and subscales of the Multidimensional Assessment of Interoceptive Awareness (MAIA) and the Multidimensional Impacts of Movement Scale (MIMS). The Trust subscale from MAIA was excluded from analysis because all participants reported the same score. An alpha value of 0.05 was utilized to assess statistical significance. Bonferroni corrections were utilized where appropriate (e.g., when a psychological assessment had several subscales).

To examine condition-dependent differences in EEG spectral dynamics, we analyzed PSD data obtained from seven independent components across five behavioral experiences: pre-dance resting state, watching dance, choreography, improvisation, and post-baseline resting state. For each component and condition, frequency peaks were identified within canonical bands (theta, alpha, beta, gamma), and their corresponding power values were extracted. These peak-level data were aggregated to compute, for each band and condition, (1) the number and proportion of components expressing a peak and (2) the total peak power (summed linear and log-transformed values). Statistical comparisons were performed at the component level using nonparametric permutation tests. First, omnibus permutation tests were conducted per frequency band to assess whether the proportion of components with peaks (binary outcome) or total peak power (continuous outcome) differed across the five conditions. Next, planned pairwise contrasts compared each condition to the pre-baseline using paired permutation tests (10,000 iterations, components treated as exchangeable units). This approach allowed inference on within-subject spectral changes without parametric assumptions, providing robust estimates of both the presence and magnitude of oscillatory peaks across behavioral experiences.

In regard to the FC data, to test pre–post change without imposing normality/sphericity assumptions and to retain all edge-level information in this case series design, we conducted two-sided label-permutation tests separately within each band × region combination (12 tests total). For each test, the statistic was the mean difference in connectivity (post − pre) across edges, and the null distribution was obtained by randomly permuting the pre/post labels 5,000 times while preserving sample sizes. We report the permutation *p*-value (*p*_perm_) and control familywise error across the 12 comparisons using Holm’s method (α = .05). For interpretability, we also report Cohen’s *d* (using the pooled edge-wise standard deviation) and 95% bootstrap confidence intervals for the mean change (percentile method, 5,000 resamples). Inference pertains to the edge distributions within this individual (edges treated as exchangeable within region type), not to a population of participants. PSD and FC data analyses were performed in Python (Anaconda; pandas, SciPy permutation_test, statsmodels for multiple-testing).

## Supplementary Material

Supplementary Files

This is a list of supplementary files associated with this preprint. Click to download.

- SupplementaryInformationScientificReports.docx

## Figures and Tables

**Table 1.** Construct validity and internal consistency of flow scales. Four scales were used to assess flow associated with general dance (dispositional) and a specific performance the night before survey administration (flow state). Separate confirmatory factor analyses were conducted for each scale and fit with specified datasets. Hypothesized models for all scales were generally a good fit for their respective datasets and reliable. Reports metrics include: Chi-Square value (χ^2^), Degrees of Freedom (df), Comparative Fit Index (CFI), Non-Normed Fit Index (NNFI), Root Mean Square Error of Approximation (RMSEA), Standardized Root Mean Square Residual (SRMR), and Cronbach’s ☒ (Reliability).

Flow Scale	Dataset	χ^2^	df	CFI	NNFI	RMSEA	SRMR	Reliability
Short Dispositional Flow (S. A. Jackson et al., 2008)	Item-identification sample (S. A. Jackson & Eklund, 2002)	66.89	27	0.99	0.98	0.05	0.03	0.81
Core Dispositional Flow (Martin & Jackson, 2008)	School students’ extracurricular activities (Martin & Jackson, 2008)	590.18	35	0.98	0.97	0.08	0.03	0.91
Short Flow State (S. A. Jackson et al., 2008)	Item-identification sample (S. A. Jackson & Eklund, 2002)	74.13	27	0.97	0.97	0.05	0.03	0.77
Core Flow State (Martin & Jackson, 2008)	Australian netball and volleyball players’ sport (Martin & Jackson, 2008)	124.79	35	0.97	0.96	0.11	0.05	0.92

**Table 2.** Final Datasets Included for Analysis. EEG data was segmented into the experiences listed above. None of these segments overlapped in time. There were moments where participants either watched or danced during the cypher and battle. Our final analyses averaged metrics of interests across experiences for watching, choreographed, and improvised dance. A significant portion of Participant 2’s datasets were excluded from analysis because of poor data quality.

Subject	Experience	Categorization
**Participant 1**	Pre-Baseline	Pre-Baseline
	Watching Cypher - Part 1	Watching Dance
	Watching Cypher - Part 2	Watching Dance
	Watching Participant 2 Dance during Battle - Part 1	Watching Dance
	Watching Participant 2 Dance during Battle - Part 2	Watching Dance
	Synchronous Choreography - Song 1	Choreographed Dance
	Synchronous Choreography - Song 2	Choreographed Dance
	Dancing during Battle	Improvised Dance
	Mirroring Freestyle	Improvised Dance
	Dancing during Cypher	Improvised Dance
	Post-Baseline	Post-Baseline
**Participant 2**	Pre-Baseline	Pre-Baseline
	Watching Cypher - Part 1	Watching Dance
	Watching Cypher - Part 2	Watching Dance
	Watching Participant 1 during Cypher	Watching Dance
	Post-Baseline	Post-Baseline

## Data Availability

All code used for analyses and plots are publicly available on GitHub at https://github.com/embodiedbrainlab/memphis-jookin-flow/tree/main. The data that support the findings of this study are available on request from the corresponding author. The data are not publicly available due to privacy or ethical restrictions.

## Key Points

- Dance enhances flow, interoceptive awareness, and mind-body integration.
- Theta (4–8 Hz) activity is present during dance across a variety of brain regions.
- Dance increases resting-state functional connectivity
